# Beneficial or detrimental activity of regulatory T cells, indoleamine 2,3-dioxygenase, and heme oxygenase-1 in the lungs is influenced by the level of virulence of *Mycobacterium tuberculosis* strain infection

**DOI:** 10.3389/fcimb.2023.1105872

**Published:** 2023-05-22

**Authors:** Vasti Lozano-Ordaz, Yadira Rodriguez-Miguez, Angel E. Ortiz-Cabrera, Sujhey Hernandez-Bazan, Dulce Mata-Espinosa, Jorge Barrios-Payan, Rafael Saavedra, Rogelio Hernandez-Pando

**Affiliations:** ^1^ Experimental Pathology Section, Department of Pathology, National Institute of Medical Sciences Nutrition Salvador Zubiran, Mexico City, Mexico; ^2^ Immunology Deparment, Biomedical Research Insitute, National Autonomous University of Mexico (UNAM), Mexico City, Mexico

**Keywords:** tuberculosis, immunoregulation, T regulatory cells, IDO, HO-1

## Abstract

Tuberculosis (TB) caused by the complex Mycobacterium tuberculosis (Mtb) is the main cause of death by a single bacterial agent. Last year, TB was the second leading infectious killer after SARS-CoV-2. Nevertheless, many biological and immunological aspects of TB are not completely elucidated, such as the complex process of immunoregulation mediated by regulatory T cells (Treg cells) and the enzymes indoleamine 2,3-dioxygenase (IDO) and heme oxygenase 1 (HO-1). In this study, the contribution of these immunoregulatory factors was compared in mice infected with Mtb strains with different levels of virulence. First Balb/c mice were infected by intratracheal route, with a high dose of mild virulence reference strain H37Rv or with a highly virulent clinical isolate (strain 5186). In the lungs of infected mice, the kinetics of Treg cells during the infection were determined by cytofluorometry and the expression of IDO and HO-1 by RT-PCR and immunohistochemistry. Then, the contribution of immune-regulation mediated by Treg cells, IDO and HO-1, was evaluated by treating infected animals with specific cytotoxic monoclonal antibodies for Treg cells depletion anti-CD25 (PC61 clone) or by blocking IDO and HO-1 activity using specific inhibitors (1-methyl-D,L-tryptophan or zinc protoporphyrin-IX, respectively). Mice infected with the mild virulent strain showed a progressive increment of Treg cells, showing this highest number at the beginning of the late phase of the infection (28 days), the same trend was observed in the expression of both enzymes being macrophages the cells that showed the highest immunostaining. Animals infected with the highly virulent strain showed lower survival (34 days) and higher amounts of Treg cells, as well as higher expression of IDO and HO-1 one week before. In comparison with non-treated animals, mice infected with strain H37Rv with depletion of Treg cells or treated with the enzymes blockers during late infection showed a significant decrease of bacilli loads, higher expression of IFN-g and lower IL-4 but with a similar extension of inflammatory lung consolidation determined by automated morphometry. In contrast, the depletion of Treg cells in infected mice with the highly virulent strain 5186 produced diffuse alveolar damage that was similar to severe acute viral pneumonia, lesser survival and increase of bacillary loads, while blocking of both IDO and HO-1 produced high bacillary loads and extensive pneumonia with necrosis. Thus, it seems that Treg cells, IDO and HO-1 activities are detrimental during late pulmonary TB induced by mild virulence Mtb, probably because these factors decrease immune protection mediated by the Th1 response. In contrast, Treg cells, IDO and HO-1 are beneficial when the infection is produced by a highly virulent strain, by regulation of excessive inflammation that produced alveolar damage, pulmonary necrosis, acute respiratory insufficiency, and rapid death.

## 1 Introduction

*Mycobacterium tuberculosis* (MTB), the etiological agent of human tuberculosis (TB) has latently infected one-fourth of the world’s population; however, only 10% of this population will develop the active disease at some point in their lives. In 2021, there were 10.6 million new cases and 1.6 million deaths due to TB; it is the 13th leading cause of death and the second leading infectious killer after COVID-19 (above HIV/AIDS) (Global Tuberculosis reporT 2022, 2022). In an efficient encounter, macrophages engulf MTB in a phagosome and later destroy them by enzymatic lysis (Collins and Kaufmann, 2001). MTB antigens are processed and presented to CD4+ and CD8+ T cells, triggering a protective Th1 response with the production of pro-inflammatory cytokines, such as IL-2, IFN-γ, IL-12, IL-8, and TNF-α (Ladel et al., 1995). Several mechanisms of immune evasion by mycobacteria can impair this Th1 response and cytokines such as IL-4, IL-5, IL-6, IL-10, IL-13, and TGF-β increase their production during progressive disease with a detriment to the protective response (Ladel et al., 1995; Behar et al., 2010). Virulent strains of MTB evade apoptosis and cause necrosis of infected alveolar macrophages (AM), favoring bacilli survival and dissemination to other macrophages (Meena and Rajni, 2010). MTB can also induce immune regulatory responses by activating Treg cells (CD4+CD25+FoxP3+) and their functions (Scott-Browne et al., 2007).

Treg cells are CD4+ lymphocytes that constitutively express the CD25 marker (α-chain of the IL-2 receptor) and FoxP3 transcription factor (Hori and Sakaguchi, 2004). These cells suppress effector immune cells by decreasing their proliferative capacity, antibody production, and cytokine secretion, for instance, FoxP3 downregulates IFN-γ, IL-4, and IL-2 (Hori and Sakaguchi, 2004; Bettelli et al., 2005). Studies in humans with active and latent TB infection show a higher number of Treg cells than non-infected controls, and patients infected with drug-resistant MTB strains show an increase of Treg cells in peripheral blood, compared with healthy subjects (Scott-Browne et al., 2007; Wergeland et al., 2011; Arram et al., 2014). After treatment of patients with TB, the Treg cell number fall, but naïve Treg cell recovery was similar to healthy controls (Zewdie et al., 2016). The number of Treg cells is increased in the infection sites and local lymph node and spleen; during active TB, this increase is more pronounced in mice infected with H37Rv MTB for prolonged periods of infection (Churina et al., 2012). Treg cells can delay the priming of effector CD4+ cells and their recruitment at the lung, prolonging the initial phase of bacterial expansion (Shafiani et al., 2010).

Another system for the immune response regulation is mediated by the enzymes indoleamine 2,3-dioxygenase (IDO) and heme oxygenase 1 (HO-1). IDO catalyzes the degradation of the essential amino acid tryptophan to N-formylkynerunine produced by dendritic cells (DCs) and macrophages, mainly induced by IFN-γ, TGF-α and β, CTLA4, and lipopolysaccharide (Divanovic et al., 2012). IDO activity decreases the production of IL-2 and therefore diminishes the proliferation of T lymphocytes; also, its metabolites can lead effector cells to apoptosis and polarize naïve cells to Treg cells (Filippini et al., 2012). In addition, TB patients with a higher amount of IDO had a worse prognosis and lower survival (Suzuki et al., 2012). Regarding HO, three isoforms of this enzyme, HO-1, HO-2, and HO-3 catalyze the degradation of heme, and their expression varies in different cell types and tissues (Wagener et al., 2003). HO-1 is the most relevant isoform for immune-regulation due to its high anti-inflammatory action that is mediated through the degradation products, such as carbon monoxide (CO), biliverdin/bilirubin, and ferritin induced by iron. CO promotes vasodilation, inhibits platelet aggregation, and suppresses cytokine production, while the other two by-products have also potent immunological properties (Fei et al., 2012). HO-1 is a determining factor in the maturation of DC; its presence promotes the production of anti-inflammatory cytokines such as IL-10 and TGF-β while decreasing the production of IL-2 (Schumacher et al., 2012). People with latent MTB infection have an increased expression of HO-1 compared with uninfected subjects, although not much is known about its role in TB (Andrade et al., 2014).

When BALB/c mice are infected with the reference mild virulence strain H37Rv, there is an initial cell-mediated immune response with a high number of activated macrophages and expression of Th1 cytokines such as IFN-γ and IL-12, which peak at 21-day post-infection that efficiently control bacterial growth (Hernandez-Pando et al., 1996). Then, 1 month after infection, there is a progressive immune-regulation mediated by Th-2 cells and high production of anti-inflammatory cytokines, such as IL-10 and TGF-β, that in coexistence with a decrease in the production of Th1 cytokines, progressive pneumonia, and high bacillary load, produce animal’s death (Hernández-Pando et al., 2008). In this model, there is also a biphasic increase in the expression of HO-1 and FoxP3 the distinctive transcription factor of Treg cells (Hernández-Pando et al., 2008). In contrast, strain 9005186, which was isolated from an epidemic burst in the South of Mexico, induced in BALB/c mice rapid death (Hernández-Pando et al., 2012). Infected animals with this strain started to die after 3 weeks and by the 5th week, all the animals died, in coexistence with high bacterial growth that peak at day 21 and significantly more pneumonia than H37Rv infection. Mice infected with a high dose of strain 9005186-showed IFNγ delayed expression and high but transient TNFα expression (Marquina-Castillo et al., 2009). Thus, this strain is more virulent than H37Rv and does not induce a protective immune response in this animal model. With the aim to study immune regulation during the infection with mild and highly virulent MTB, we compared the kinetics of Treg cells, IDO, and HO-1 during the infection in BALB/c mice with H37Rv or 5186 strain. Then, we studied the evolution of the disease after Treg cell elimination by the administration of cytolytic monoclonal antibodies and blocking IDO or HO-1 activity with the administration of specific antagonist drugs.

## 2 Materials and methods

### 2.1 Mycobacterium tuberculosis growth

All procedures were performed in a laminar flow cabinet in a biosafety level III laboratory. Mild virulent strains H37Rv and highly virulent clinical isolate 9005186 (5186) were grown in Middlebrook 7H9 broth (Difco, Detroit, MI, USA) supplemented with 10% ADC (albumin/dextrose/catalase) (Difco, Detroit, MI, USA), 0.05% Tween 80 and 0.5% glycerol to mid-exponential phase before freezing at −70°C. Frozen stocks were freshly thawed and reconstituted at room temperature at the time of infection. A vial of stock bacilli was diluted in PBS to a final concentration of 2.5 × 10^5^ colony-forming units (CFUs) in 100 μl of PBS. Before infection, each stock vial was plated for CFU enumeration to reconfirm the standard concentration of the inoculum. The CFU concentration of stock vials was calculated by serial dilution and plating in multiple replicates on Middlebrook 7H11 agar (Difco, Detroit, MI, USA) containing 0.5% glycerol and 10% OADC (Oleic albumin/dextrose/catalase) growth enrichment. Plates were incubated for 21 days at 37°C before the determination of CFU.

### 2.2 Experimental model of progressive pulmonary tuberculosis

Pathogen-free male BALB/c mice, 6–8 weeks of age, were anesthetized (Sevoflurane; Abbott Laboratories, IL, USA) and infected by intratracheal (i.t.) cannulation, administering 2.5 × 10^5^ viable bacteria suspended in 100 μl of PBS. Infected mice were maintained in groups of five in cages fitted with microisolators connected to negative pressure. Animals were randomly selected in groups of five and euthanized by exsanguination on days 7, 14, 21, 28, 60, or 90 post-infection with the H37Rv strain. Mice infected with 5186 were euthanized at days 1, 3, 7, 14, 21 and 28 post-infection, there were no survivor animals after 37 days of infection. Lungs were either immediately frozen in liquid nitrogen, fixed by perfusion with absolute ethanol for histological studies or collected in RPMI to be processed immediately for flow cytometry. Two independent experiments were performed. All procedures were done in a biological security cabinet at a Biosafety level III facility. All the animal work was carried out according to the guidelines and approval of the Ethical Committee for Experimentation on Animals of the National Institute of Medical Sciences and Nutrition (INCMNSZ) in Mexico City, permit number CINVA 224.

### 2.3 Assessment of colony-forming units

Lungs were frozen for at least 24h at −70°C. Frozen lungs were disrupted in a Polytron homogenizer (Kinematica; Lucerne, Switzerland) in sterile tubes containing 1 ml of PBS-Tween 80 0.05%. Four dilutions of each homogenate were spread onto duplicate plates containing Bacto Middlebrook 7H10 agar (Difco BD, Sparks, MD, USA) enriched with OADC (Oleic albumin/dextrose/catalase. Difco). The incubation time of the plates was 21 days, and data points are the means of four animals.

### 2.4 Flow cytometry

The right lungs from three mice were processed immediately after euthanizing. Lungs were immersed in 2 ml of RPMI (Gibco Life Technologies, Grand Island, NY, USA) with 1 mg/ml of collagenase II (Gibco Life Technologies, Grand Island, NY, USA) and incubated for 1h at 37°C. After that, lungs were disaggregated by passing through needles of different caliber (18G and 21G), and the obtained cells were washed with PBS/albumin 2% and filtered in a 70-μm strainer (Beckton Dikinson, USA). 5 × 10^6^ cells from each mouse were stimulated for 6h with Cell Stimulation Cocktail (Tonbo Bioscience, San Diego, CA, USA). Later 1 × 10^6^ cells were harvested and washed with PBS and blocked with 1 μl of purified anti-mouse CD16/CD32 (Fc Shield) (Tonbo Bioscience, San Diego, CA, USA). After that, cells were stained with Ghost Dye 450 (Tonbo) to separate dead/living cells and then were stained with anti-CD3 AF, anti-CD4/BV450 and anti-CD25/BB515, all diluted 1:100 in PBS. Finally, intracellular labeling to detect FoxP3/PE was performed with specific antibodies and diluted 1:100 in FixPerm (eBioscience, USA). Samples were analyzed in BD LSRFortessa™ (BD LSRTFortessa, USA). Data were analyzed by FlowJo™ v.10 (FlowJo v.10, USA). First, the lymphocytes zone was selected from FSC *vs.* SSC gate, and then alive cells were selected for gated CD3 *vs.* CD4. Finally, the percentage of CD25+FoxP3+ cells was obtained from CD3+CD4+ lymphocytes (**Supplementary Figure S1**).

### 2.5 Immunohistochemistry and morphometry

The left lungs from four mice in two independent experiments were immersed in absolute ethanol for 2 days, and parasagittal sections were taken through the hilum. Tissue samples were dehydrated, cleared, and embedded in paraffin. Lung paraffin blocks were sectioned at 4 μm and stained with hematoxylin and eosin (H&E) or phosphotungstic acid/hematoxylin stain for histological analysis. The percentage of lung area affected by pneumonia was measured using a Leica Q500/W Image Analysis System (Milton Keynes, UK).

The same paraffin blocks were used for immunohistochemistry. Lung sections 5 µm were mounted on silane-covered slides. After deparaffination and rehydration, peroxidase activity was blocked by incubation with 0.3% H_2_O_2_ in absolute methanol. Lung sections were incubated overnight at room temperature with polyclonal rabbit antibody against HO-1 (Aviva System Biology, USA) or polyclonal goat antibody against IDO-1 (LS Bio, USA) both diluted 1:200, monoclonal rabbit anti FoxP3 (R&D System, USA) diluted 1:20 in PBS, monoclonal rat anti–IL-17 (Santa Cruz, CA, USA) diluted 1:100, polyclonal mouse anti–PECAM-1(CD31), polyclonal mouse anti-GPIV (CD36), polyclonal mouse anti–P-selectin (CD62P), polyclonal anti–VCAM-1 (CD106) all diluted 1:200, polyclonal mouse anti–IL-1 (Santa Cruz, CA, USA) 1:100, polyclonal anti–TGF-β (Santa Cruz, CA, USA) diluted 1:100, polyclonal anti–IL-4 (Santa Cruz, CA, USA), polyclonal anti–TNF-α (Santa Cruz, CA, USA), and polyclonal Ly6G (Santa Cruz, CA, USA) diluted 1:250. Followed by incubation with Goat-on-Rodent HRP-polymer (BioCare, USA), anti-rabbit IgG (Vectastain, Vector Laboratories, USA) or biotinylated antimouse IgG. Bound antibodies were detected with diamino-bencidin (ImmPACT DAB, Vector Laboratories). For semi-quantification, five random fields of each sample were analysed, and the total number of cells and percentage of immunostained cells were determined using an automated image analyzer (QWin Leica, Milton Keynes, UK) to acquire images and ImageJ 1.52a software (National Institute of Health) for positive cell counting.

### 2.6 Real-time polymerase chain reaction analysis of enzymes and cytokines expression

Three lungs from each of the groups were used to isolate mRNA using the RNAeasy minikit (Qiagen, Valencia, CA, USA). The quality and quantity of RNA were evaluated through spectrophotometry (260/280) and on agarose gels. Reverse transcription of the mRNA was performed using 100 ng RNA, oligo (dT), and the Omniscript kit (Qiagen, Valencia, CA, USA). Real-time polymerase chain reaction (PCR) was carried out using the 7500 real-time PCR system (Applied Biosystems) and Quantitect SYBR green Mastermix kit (Qiagen, Valencia, CA, USA). Standard curves of quantified and diluted PCR products, as well as negative controls, were included in each PCR run. Specific primers for genes encoding the ribosomal protein, large P0 (RPLP0) as house-keeping gene, IL-4, IFN-ϒ, TGF-β, TNF-α, IDO, HO-1, IL-22, or IL-17 were designed using the program Primer Express (Applied Biosystem, San Francisco, CA, USA) (**Table 1**). Data are shown as copies of cytokine-specific mRNA/10^6^ copies of RLP0 mRNA.

**Table 1:** Primers sequences.

Gen	Forward primer	Reverse primer
Rplp0	5′-CTCTCGCTTTCTGGAGGGTG-3′	5′-ACGCGCTTGTACCCATTGAT-3′
IFN-γ	5′-CCTCAAACTTGGCAATACTCATGA-3′	5′-GGTGACATGAAAATCCTGCAG-3′
TNF-α	5′-AAATGGGCTCCCTCTCATCAGT-3′	5′-GATCTGAGTGTGAGGGTCTGGG-3′
IL-4	5′-ATGCCTGGATTCATCGATAAGC-3′	5′-GAGTAATCCATTTGCATGATGCTCT-3′
TGF-β	5′-GCTGATCCCGTTGATTTCCA-3′	5′-GTGGCTGAACCAAGGAGACG-3′
IDO	5′-CAGGCCAGAGCAGCATCTTC-3′	5′-GCCAGCCTCGTGTTTTATTCC-3′
HO-1	5′-GCCGAGAATGCTGAGTTCATG-3′	5′-TGGTACAAGGAAGCCATCACC-3′
IL-17	5′-TGACCCCTAAGAAACCCCCA-3′	5′-GTGGAGGGCAGACAATTCTGA-3′
IL-22	5′-TCAACTTCACCCTGGAAGACG-3′	5′-TGAGTTTGGTCAGGAAAGGCA-3′

### 2.7 Inhibition of immunoregulation

In order to evaluate the contribution of immune regulatory response, Treg cells were deleted using 200 μg of anti-CD25 mAb (PC61 clone), and as a negative control, isotype IgG antibody was administrated by intraperitoneal (i.p.) route, 1 day before infection in H37Rv or 5186 infected mice or on day 17 after infection with strain 5186 (**Supplementary Figure S2**), as previously described (Tenorio et al., 2010).

The activity of the regulatory enzymes was inhibited using specific blocker drugs. 1-methyl-D, L-tryptophan (1-MT) 8 mg/kg was administrated by oral route, dissolved in 100 μl of 0.5% Tween 80/0.5% Methylcellulose, v/v in distilled water was administrated to block IDO, and 50 μmol/kg of zinc protoporphyrin-IX(ZnPp) administered by intraperitoneal route, diluted in 0.05% DMSO v/v in PBS, to suppress HO-1. Control groups received the respective vehicle. Inhibitors were administrated daily in separate groups of H37Rv-infected mice to evaluate during the acute or progressive phase. In 5186-infected mice, both inhibitors were administered daily for 1 week, starting 17 days after infection. (**Supplementary Figure S2**).

### 2.8 Statistical analysis

All data were analyzed with GraphPad Prism 6 (GraphPad Software, Inc., La Jolla, CA). Statistical significance was defined using two-way analysis of variance (ANOVA) or one-way ANOVA test according to data characteristics, for comparing experimental groups at a 95% confidence interval (*p* < 0.05).

## 3 Results

### 3.1 Kinetics of Treg cells, indoleamine 2,3-dioxygenase, and heme oxygenase 1 during pulmonary tuberculosis produced by mild and highly virulent strains

**Figure 1:**
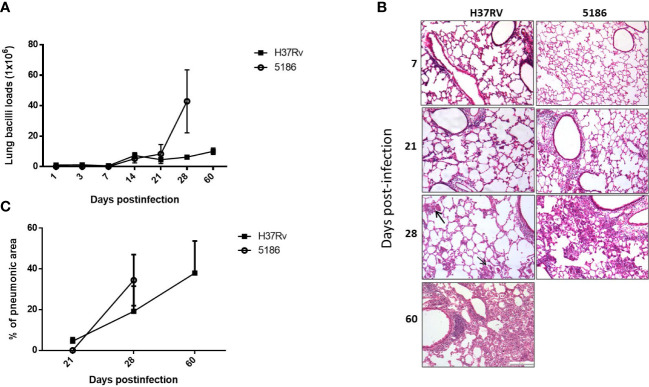
Comparison of pulmonary bacillary loads and tissue damage in BALB/c mice infected with mild or hypervirulent M. tuberculosis strains. **(A)** Male mice infected by intratracheal route with high dose of mild virulent H37Rv strain or highly virulent 5186 strain were euthanized in the indicated days, and the right lungs of three mice were used to determine bacillary loads by colony forming units count. **(B)** Left lungs were perfused with absolute ethylic alcohol and used to get tissue sections to determine the percentage of area affected by pneumonia with automated morphometry. Asterisks represent statistical significance (*p* < 0.5). **(C)** Representative micrographs of the lungs after the indicated days of infection, similar inflammatory infiltrate around blood vessels and airways was produced by either mycobacterial strains after 1 and 3 weeks of infection, small and focal areas of pneumonia were observed after 1 month of infection with mild virulent H37Rv strain (arrows), while extensive pneumonia at the same time point was seen in animals infected with highly virulent 5186 strain that was similar after two months of infection with strain H37Rv (all micrographs 200× magnification, stain with haematoxylin/eosin).

Mice infected with mild virulent H37Rv strain showed a progressive increase of bacillary loads from day 14 post-infection, raising its peak at day 60, which correlated with progressive and extensive pneumonia that affects more than 50% of lung surface area at this time of infection (**Figure 1**). Mice infected with strain 5186 showed similar bacillary loads than infected mice with strain H37Rv until day 21, and then a striking sudden increase of bacillary burdens and pneumonia was seen in mice infected with the highly virulent strain at day 28 after infection (**Figure 1**).

**Figure 2:**
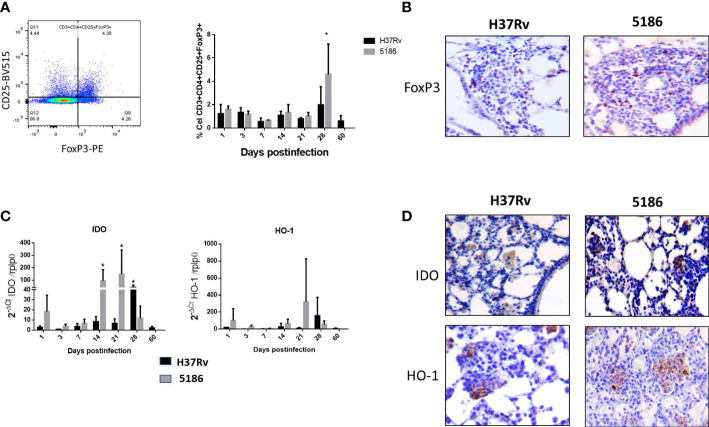
Comparative kinetics of Treg cells during pulmonary infection with mild or highly virulent M. tuberculosis strains. **(A)** Left panel shows the cytofluorometry dot distribution for the selection of CD-4/CD-25/FoxP3 cells. Right panel shows the comparative percentage of Treg cells determined by cytofluorometry in lung homogenates after the infection with mild virulent strain H37Rv (black bars) and hyper-virulent strain 5186 (gray bars). **(B)** Representative micrographs of perivascular inflammation after 28 of infection with the indicated strain, positive immunostaining of FoxP3 is observed in some lymphocytes, being more numerous in the mouse infected with the 5186 highly virulent strain. **(C)** Groups of three mice infected with mild virulent strain H37Rv (black bars) or highly virulent strain 5186 (gray bars) were euthanized at the indicated days of infection, their lungs were homogenized and the gene expression of IDO and HO-1 was determined by RT-PCR. Asterisks represent statistical significance (*p* < 0.5). **(D)** Representative micrographs of IDO and HO-1 detection by immunohistochemistry after 28 days of infection with the indicated strain, immunostained cells are macrophages located in pneumonia areas, being more numerous in the infection with 5186 strain (40× magnification).

The immune regulatory response was evaluated by the percentage of Treg cells (CD3+CD4+CD25+FoxP3+) from a total of CD3+CD4+ live cells (**Supplementary Figure S1**). Animals infected with strain H37Rv showed a stable low percentage of regulatory T cells, except at day 28 when a non-significant increase was seen (**Figure 2A**). Similar kinetics was observed in mice infected with the highly virulent strain 5186, but at day 28 of infection was also the peak of the percentage of Treg cells being twofold higher compared with the previous days and the same day of infection with H37Rv (**Figure 2A**). By immunohistochemistry in lung sections of 28 days of infection, Treg cells were found in the inflammatory infiltrate around blood vessels and airways, as well as in granulomas and pneumonic areas, being more abundant in mice infected with the highly virulent strain (**Figure 2B**).

IDO and HO-1 exhibited similar gene expression kinetics, showing an increase since day 1 post-infection and raising its peak at day 28 post-infection in mice infected with the H37Rv strain. Animals infected with hypervirulent strain 5186 showed maximal expression on days 14 and 21 for IDO and on day 21 for HO-1 (**Figure 2C**). According to these results, IDO and HO-1 expression in the lungs were studied by immunohistochemistry at 28 days post-infection with either strain. Both IDO and HO-1 were essentially detected in pneumonic areas, particularly strong staining was detected in macrophages (**Figure 2D**).

### 3.2 The effect of depletion of regulatory T cells during H37Rv and 5186 *Mycobacterium tuberculosis* strains infection

To study the participation of Treg cells during the course of the disease, these cells were depleted by the administration of monoclonal antibody anti-CD25 (PC61 clone). To confirm the antibody effect, percentages of T cells were determined at days 7 and 21 after antibody administration in mice infected with H37Rv. Treated mice showed a significant fivefold lesser Treg cells percentage compared with controls on day 7, while on day 21, similar percentages between control and treated mice were observed. No significant differences in the percentages of Th1/Th2 cells were found in treated mice (**Supplementary Figure S3**).

**Figure 3:**
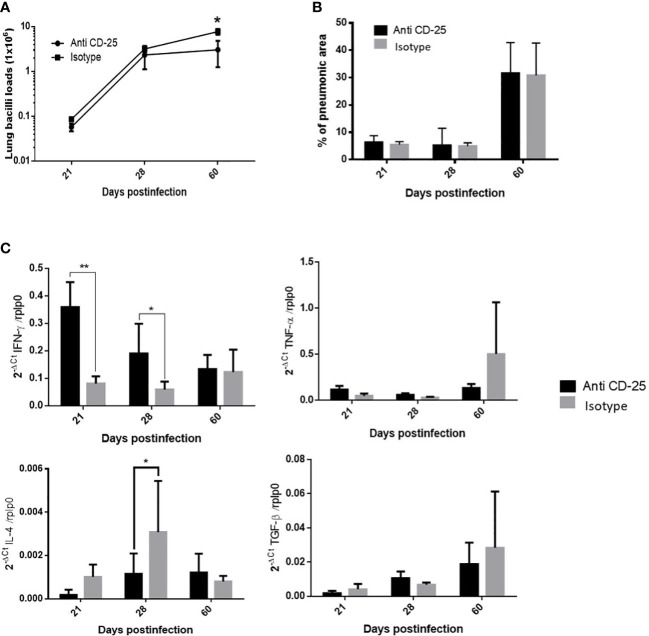
The effect of Treg cells depletion in BALB/c mice infected with mild virulent M. tuberculosis strain H37Rv. **(A)** Treg cells were depleted by the intraperitoneal administration of 200 μg of monoclonal antibodies anti-CD25 mAb (PC61 clone), 1 day before infection with mild virulent strain H37Rv in BALB/c mice, while control infected mice received the same amount of irrelevant isotype antibodies by the same route, groups of three mice were euthanized at the indicated days and their right lungs were used to determine bacillary loads by colony forming units quantification. **(B)** Left lungs were perfused with ethylic alcohol to obtain histological sections that were used to determine the percentage area affected by pneumonia by automated morphometry. **(C)** Frozen lungs from three mice per group in the indicated days of infection were used to isolate total RNA, which was used to determine the expression of pro-inflammatory (IFN-γ and TNF-α) and anti-inflammatory (IL-4 and TGF-β) cytokines by RT-PCR. Asterisks represent statistical significance (*p* < 0.5).

Considering that Treg cells are constantly present during the course of H37Rv strain infection with minimal fluctuations, anti-CD25 antibodies were administrated 1 day before infection; the control group received an irrelevant isotype IgG antibody. In comparison with control mice, a significant decrease in bacillary load was seen in animals treated with the antibodies at day 60 post-infection (**Figure 3A**). The expression of significant cytokines in the immune response during pulmonary TB (IFN-γ, IL-4, TNF- α, and TGF-β) was determined. Treated animals showed an increase of IFN-γ that was significant on days 21 and 28, while IL-4 shows a significant decrease on day 28 post-infection. Due to the large SD, treated mice showed a non-significant increase in TNF-α at days 21 and 28. No differences were observed in TGF-β expression, while the extension of pneumonic areas was similar between treated and control animals (**Figures 3B, C**).

**Figure 4:**
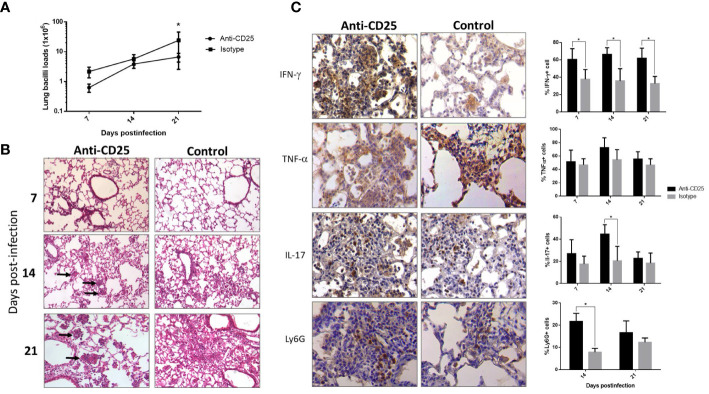
The effect of Treg cells depletion during early phase, in BALB/c mice infected with highly virulent M. tuberculosis strain. **(A)** Treg cells were depleted by the intraperitoneal administration of 200 μg of monoclonal antibodies anti-CD25 mAb (PC61 clone) 1 day before infection with highly virulent strain 5186 in BALB/c mice, while control infected mice received the same amount of irrelevant isotype antibodies by the same route, groups of three mice were euthanized at 7, 14, and 21 days of infection and their right lungs were used to determine bacillary loads by colony forming units quantification. **(B)** Representative comparative micrographs of the lungs from anti CD-25 mAb-treated and control-infected mice; at day 7 of infection, there is slight higher perivascular inflammation in mouse treated with the CD-25 antibodies. After 2 weeks of infection, treated mouse show more well-formed granulomas (arrows). After 3 weeks of infection, treated animal show in the center of many alveoli conglomerates of inflammatory cells (arrows) that correspond to alveolitis, while control mouse shows extensive pneumonia. **(C)** Representative comparative immunohistochemistry micrographs of the lungs from anti CD-25 mAb treated and control; after 21 days of infection, there are more immunostained cells of the indicated cytokine in granulomas from treated than control animals, perivascular inflammation also shows more neutrophils positive to the marker Ly6G in treated mice. This was confirmed by semi-quantitative automated morphometry (right panel). Asterisks represent statistical significance (*p* < 0.5).

Mice infected with the highly virulent strain 5186 also showed a constant percentage of pulmonary Treg cells during the course of the infection, except at day 28 when the significantly higher increase was determined in coexistence with extensive pneumonia and high bacillary loads. Thus, mice infected with this highly virulent strain received the anti-CD25 1 day before infection or at day 17 post-infection in order to get efficient Treg depletion at day 28 of infection. When Treg cells were depleted since day 1 of infection, a decrease in bacilli load was seen at day 21 postinfection when compared with control mice (**Figure 4A**). In comparison with control mice, histopathology analysis showed in treated mice more inflammatory infiltrate constituted by macrophages, lymphocytes, and numerous neutrophils around blood vessels and airways, as well as more well-formed granulomas after 2 weeks of infection. At day 21 of infection, treated mice showed groups of macrophages with lymphocytes congregated in the center of some alveoli that correspond to areas of alveolitis, while control mice showed extensive pneumonia (**Figure 4B**). Neutrophil recruitment was confirmed by immunohistochemistry, showing a higher number of Ly6G positive cells in treated mice compared with control (**Figure 4C**). Because the inflammatory response in treated mice was different than in control animals, the production of cytokines was evaluated by immunohistochemistry and automated morphometry, which showed more immune stained cells to IFN-γ, TNF-α, and IL-17 in the perivascular inflammatory infiltrate and granulomas of treated mice than in control animals (**Figure 4C**).

**Figure 5:**
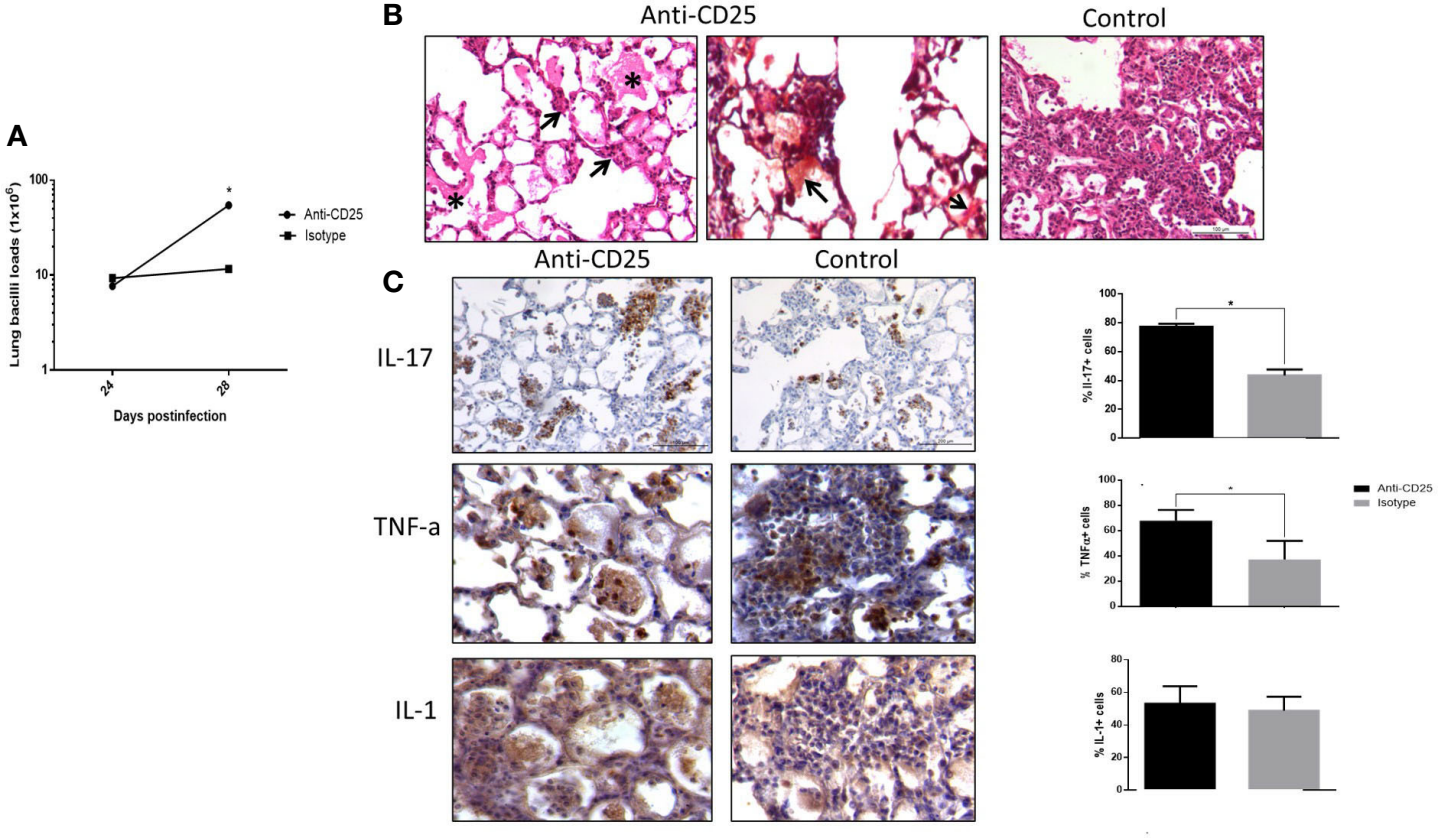
The effect of Treg cells depletion in BALB/c mice infected with highly virulent M. tuberculosis strain. **(A)** Treg cells were depleted by the intraperitoneal administration of 200 μg of monoclonal antibodies anti-CD25 mAb (PC61 clone) at day 17 of infection with highly virulent strain 5186 in BALB/c mice, while control-infected mice received the same amount of irrelevant isotype antibodies by the same route, groups of three mice were euthanized at 24 and 28 days of infection and their right lungs were used to determine bacillary loads by colony forming units quantification. There is a significant increase of bacillary burdens at 28 days of infection in treated animals. **(B)** Representative comparative micrographs of treated and control animals after 28 days of infection, left figure correspond to treated mouse that shows eosinophilic hyaline membranes on the surface of alveolar walls (asterisk) and chronic inflammatory infiltrate in the alveolar capillary interstitium (arrows), which characterize diffuse alveolar damage. Centre figure show a section from the same treated mouse stained with Phosphotungstic acid haematoxylin that show positivity, red staining (arrows) of the hyaline membranes confirming that they are constituted by fibrin, while control non-treated infected mice show extensive pneumonia (right figure). **(C)** Representative micrographs of cytokines detection by immunohistochemistry in the lungs of mice after 28 days of infection with highly virulent M. tuberculosis strain and treated or not with anti-CD25 monoclonal antibodies, treated mouse shows more immunostained cells of the indicated cytokines in areas of alveolar damage than in pneumonic areas of control animal, which is confirmed by automated semiquantitative morphometry (right panel). Asterisks represent statistical significance (*p* < 0.5).

**Figure 6:**
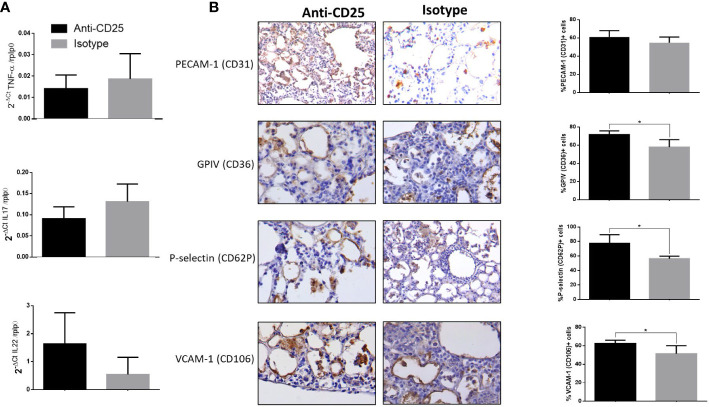
Detection of adherence molecules by immunohistochemistry and expression of cytokines in mice infected with hyper virulent M. tuberculosis strain. **(A)** Groups of mice infected with highly virulent strain 5186 and treated or not with monoclonal antibodies anti-CD25 mAb (PC61 clone) were euthanized after 28 days of infection, and their right lungs were used to determine the expression of IL-22, IL-17, and TNF-α by RT-PCR. **(B)** Representative immunohistochemistry micrographs of the indicated molecules detected after 28 days of infection, in areas of diffuse alveolar damage from treated animals with anti-CD25 monoclonal antibodies and pneumonic areas from control non-treated animals. All the determined adhesion molecules are more expressed in treated mice than in control animals, which is confirmed by automated semiquantitative morphometry (right panel) determining in five fields for each sample the number and percentage of positive cells. Asterisks represent statistical significance (*p* < 0.5).

Infected mice with highly virulent TB strain and treated with anti-CD25 antibodies at day 17 of infection showed similar bacillary loads and pneumonia to the control group after 1 week of Treg cell depletion (24 days postinfection) (**Figure 5A**). However, survival decreased considerably in anti-CD25–treated mice before day 28 post-infection and survivor animals were very sick; so, for humane reasons, all the animals were euthanized on day 28 after infection. At this time point, animals with Treg cell depletion showed fivefold more CFU than control mice (**Figure 5A**). The lungs of control mice showed extensive pneumonia, while animals treated with monoclonal antibodies against CD-25 exhibited diffuse alveolar damage that affect 46 ± 8% of the lung surface. Diffuse alveolar damage was characterized by the deposition of thick eosinophilic hyaline membranes on the surface of alveolar walls, in coexistence with mononuclear inflammatory cells infiltrate in the alveolar-capillary interstitium (**Figure 5B**). These histological abnormalities are quite similar to the common tissue damage induced by severe acute viral infection. Diffuse alveolar damage was confirmed by the presence of fibrin in these hyaline membranes demonstrated by histochemistry (phosphotungstic acid/hematoxylin staining) (**Figure 5C**). An imbalance of Th17/Treg cells can induce acute lung injury with elevated expression of IL-17 and IL-22, as well as pro-inflammatory cytokines such as TNF-α and IL-1-β (Zhang et al., 2016; Stephen et al., 2018; Wang et al., 2019). In agreement with this, areas with hyaline membranes exhibited numerous lymphocytes with intense IL-17 immunostaining and macrophages showing strong reactivity to TNF-α and IL-1-β, while in non-treated mice, these cytokines were expressed in pneumonic areas. Automated morphometry confirmed the higher percentage of positive cells to IL-17 and TNF-α in treated mice compared with the control group (**Figure 5D**), but no differences were observed in the expression of IL-17 and TNF-α determined by RT-PCR in whole lungs homogenates; only IL-22 showed higher but no significant expression in treated mice compared with controls (**Figure 6A**). To evaluate changes in endothelium in areas of diffuse alveolar damage, PECAM-1, GPIV, P selectin, and VCAM-1 showed strong immunostaining in endothelial cells, alveolar epithelium and leukocytes that was confirmed by automated morphometry, which showed higher percentages of positive cells for these adherence molecules in anti-CD25–treated mice than in control animals (**Figure 6B**).

### 3.3 The effect of blocking indoleamine 2,3-dioxygenase, and heme oxygenase 1 during infection with mild virulent H37Rv or highly virulent 5186 strain

Considering that gene expression of IDO and HO-1 was detected since the beginning of the H37Rv strain infection and showed their peak at 28 days of infection, the inhibitor 1MT of IDO activity and ZnPp that block HO-1 was administrated during the second and third week of infection, and mice were euthanized at 2 months of infection. In comparison with non-treated control mice, treated animals with IDO blocker showed a non-significant decrease of pulmonary bacillary counts and lesser expression of IFN-γ, IL-4, TNF-α, and TGF-β, as well as lesser pneumonia, but these were non-significant (**Supplementary Figure S4A**). Infected animals with the H37Rv strain and treated with the HO-1 inhibitor ZnPp administered during the second and third week of the infection, and euthanizing mice after 60 days of infection showed lower bacillary loads and pneumonia, as well as higher expression of IFN-γ and lower transcription of IL-4 and TGF-β, but only the transcription of TGF-β was significant (**Supplementary Figure S5A**).

Because macrophages from pneumonic areas in late disease were the principal IDO and HO-1 immunostained cells, another experiment was performed administrating the inhibitors of these enzymes from the first month after infection and for 2 months, euthanizing mice at 60 and 90 days after infection. In comparison with control, non-treated mice, blocking IDO produced a decrease of bacilli burdens at day 60 post-infection, without significant difference in IFN-γ, TNF-α, and IL-4 expression. No changes in the pneumonic area were observed either (**Supplementary Figure S4B** **).** Blocking HO-1 administrating ZnPp during late TB induced non-significant lesser bacterial loads, while cytokines expression and pneumonic area were similar in both groups (**Supplementary Figure S5B**).

**Figure 7:**
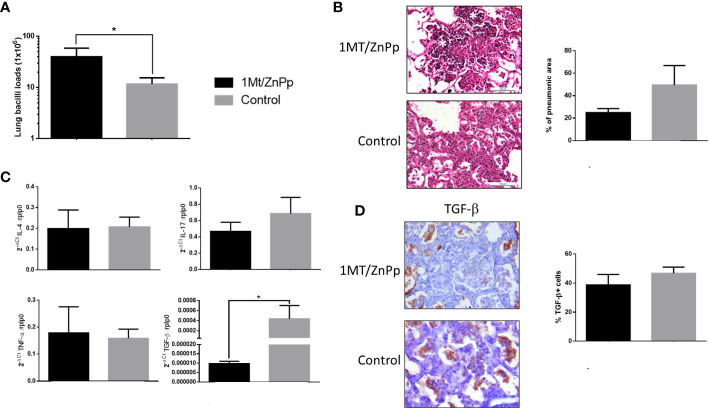
The effect of suppress IDO and HO-1 during pulmonary infection with hyper virulent mycobacteria. **(A)** Mice infected with highly virulent strain 5186 were treated or not with 1MT and ZnPp to block both IDO and HO-1 since day 17 post-infection, and groups of mice were euthanized at days 24 and 28 post-infection, right lungs were used to determine bacillary burdens. Treated mice showed significant higher bacillary loads. **(B)** Representative micrographs of control non-treated mice after 28 days of infection showed extensive pneumonia, treated mice showed also extensive pneumonia but with focal areas of necrosis manifested by cells with condensed (picnotic) or fragmented (cariorrexis) nucleus (white asterisks). In the same section was determined the percentage of pneumonic areas by automated morphometry. **(C)** The lungs of other group of mice after 28 days of infection from treated and control groups were used to isolate total RNA, and the expression of the indicated cytokines was determined by RT-PCR. Treated mice only showed significant lower expression of TGF-β. **(D)** Representative micrographs of the immunohistochemistry detection of TGF-β. Semi-quantitative analysis was performed to identified percentage of positive cells, from five fields random chosen. Asterisks represent statistical significance (*p* < 0.5).

To evaluate the role of regulatory response mediated by IDO and HO-1 during hypervirulent strain infection and due to the low number of survivor mice, animals infected with strain 5186 were treated with both 1MT and ZnPp to block IDO and HO-1 since day 17 post-infection, and control non-treated and treated mice were euthanized at day 28 post-infection. In comparison with control animals, mice treated with both 1MT and ZnPp showed higher bacillary loads at day 28 of infection (**Figure 7A**). There was a lower percentage of lung surface area affected by pneumonia in treated animals, and some of these pneumonic areas show extensive necrosis manifested by numerous cells with a condensed or fragmented nucleus and cytoplasmic disruption (**Figure 7B**). To study the inflammatory response related to necrosis in TB infection, the expression of IL-4, IL-17, TGF-β, and TNF-α was evaluated. The expression of cytokines determined at day 28 of infection showed similar expression between control and treated groups; only significantly lower gene expression of TGF-β in treated animals than control mice was observed, and these treated mice showed lesser but no significative percentage of TGF-β–stained cells in the pneumonic areas than control mice (**Figures 7C, D**).

## 4 Discussion

A successful host immune response is generally the result of pro- and anti-inflammatory factors that are carefully tuned with the aim to eliminate the pathogen and limiting tissue damage. The tune and balance of pro and anti-inflammatory factors is a dynamic process that requires a constant relation between the pathogen and the immune cells and their cytokines production. This interaction is not always quantitative, with equal concentrations of pro- and anti-inflammatory cytokines and a similar number of immune cells, but it is more a qualitative harmonization in downstream activation and inhibition in which the pathogen is also a significant participant (Cicchese et al., 2018). Regarding TB, the participation of Treg cells in this regulatory activity and whether they are beneficial or detrimental is a recurring debate (Cardona and Cardona, 2019).

Many reports revealed that there is an increase in Treg cells, IDO, and HO-1 in patients with active or latent TB (Churina et al., 2012; Suzuki et al., 2012; Andrade et al., 2015). These high levels of immune-regulatory factors should be a consequence of intense inflammation, but it is not completely clear if these immune-regulatory factors are beneficial or deleterious for the development or severity of TB. Previously in our murine TB model using the H37Rv strain was observed a biphasic increase in the gene expression of Fox-p3, IDO, and HO-1 (Hernández-Pando et al., 2008), with high expression of these molecules after one week, followed by a decrease and an increase again after one month of infection (Hernández-Pando et al., 2008), which is the time point when in our model start pneumonia formation and there is high production of IFN-γ (Hernandez-Pando et al., 1996). It is known that Treg cells, IDO, and HO-1 increase in stress conditions and pro-inflammatory environments, mainly with high IFN-γ levels (Quinn et al., 2006; Sun et al., 2011; Schumacher et al., 2012). Treg cells, IDO, and HO-1 enzymes lead to a decrease of effector cell proliferation and induce apoptosis by diverse mechanisms, resulting in a decrease in pro-inflammatory cytokines production (Hori and Sakaguchi, 2004; Bettelli et al., 2005; Fontenot and Rudensky, 2005), which is the type of immune response produced during late infection in our murine model (Hernandez-Pando et al., 1996). Treg cells were depleted or the activity of IDO and HO-1 was inhibited since the beginning of infection with mild virulence strain H37Rv, the administration of anti-CD25 antibodies or enzyme blockers was initiated at this time because these regulatory factors started their increase since day 1 and it was relatively stable during the infection, an increase of IFN-γ expression was observed, but this was only significant with Treg cell depletion. This result is in concordance with the results of other groups that also observed an increment in pro-inflammatory cytokines and a decrease in anti-inflammatory activity when they used inhibitors of these immune regulator factors (Belkaid et al., 2002; Kohm et al., 2006; Jaron et al., 2008). Our determination of pulmonary bacilli burdens showed a significant decrease in mice infected with mild virulence H37Rv strain and depletion of Treg cells or late inhibition of IDO activity, which correlated with an increase of IFN-γ and a decrease of IL-4 that favored the protective Th1 response. Thus, the experimental decrease of Treg cells and IDO in BALB/c mice infected with mild virulence reference strain H37Rv is beneficial, which is in agreement with the previous observations that show delayed recruitment of effector cells by Treg cells in the lung during early TB (Shafiani et al., 2010), when Tregs were depleted in the early phase of infection, effector T cells migrated correctly and limit the expansion of bacilli load in progressive phase but is not enough to change the course of infection. One limitation of our work was the missed experiment of Treg cells depletion since day 28 of infection with strain H37Rv, when the peak of these cells was seen and pneumonia started its formation, we suppose that the beneficial effect of Treg cells deletion should be more evident starting at this time, because there is in the pneumonic areas Treg cells in co-existence with macrophages that exhibited IDO and HO-1 strong immunostaining. Other groups reported previously that when they depleted Treg or inhibited IDO enzyme, bacilli loads were not affected, although there was an increase in pro-inflammatory cytokines (Quinn et al., 2006; Mulley and Nikolic-Paterson, 2008; Divanovic et al., 2012), and a similar response was observed in mice that received ZnPP to inhibit HO-1 (Hou et al., 2007). In this regard, it is important to consider the complexity of these immune regulatory mechanisms; actually, they mutually modulate directly or indirectly. This is the case of nitric oxide (NO), which is produced by the enzyme inducible nitric oxidase synthase (iNOS) and is a significant mycobactericidal agent, particularly in mice (Hernández-Pando et al., ). HO-1 expression reduced iNOS activity and therefore NO production, while NO diminished IDO activity. Hence, a decrease in NO production by HO-1 activity indirectly increased IDO activity (Srisook and Cha, 2005). Another example is the close relationship between IDO expression and Treg polarization from Th0 cells and their maintenance (Sun et al., 2011); also, Treg cells induce IDO expression through CTLA-4 in DCs and macrophages (Sun et al., 2011; Efimov et al., 2012). Nevertheless, the pro-inflammatory environment is the principal inductor of the regulatory response, so that the experimental depletion of Treg or the specific inhibition of IDO or HO-1 can induce diverse mechanisms that compensate for the intervened regulatory response.

When Treg cells were depleted 1 day before infection with highly virulent strain 5186, recruitment of leukocyte cells was higher than in control mice after 1 week of infection, which correlated with a previous report that observed Treg cells delay migrations cells to the lung (Shafiani et al., 2010). Also, a higher IFN-γ production was observed at all analyzed days postinfection, as was previously described (Quinn et al., 2006; Singh et al., 2012). This was manifested in the more organized and effective inflammatory response, with well-organized granulomas and lower bacilli load, with alveolitis instead of pneumonia at day 21 postinfection in treated mice, that contrasted with control animals, that showed extensive pneumonia. A previous report has characterized the TB granuloma microenvironment and observed an important regulatory activity that includes Treg cells and IDO expression, which can decrease the efficiency of granuloma to contain mycobacteria growth (McCaffrey et al., 2022). Mice that received anti-CD25 at the beginning of infection to deplete Treg cells showed well-formed granulomas than in control mice at day 21 post-infection. Moreover, there was higher neutrophil recruitment in treated mice compared with control, associated with higher production of IL-17 that correlated with the well-known imbalance of Th17/Treg (Fletcher et al., 2009; Da Silva et al., 2015; Agrawal et al., 2018; Safar et al., 2020). All these observations may influence the decrease of lung bacilli load and inflammation in our treated mice.

Interestingly, when Treg cells were depleted after 2 weeks of infection in mice infected with a highly virulent strain 5186, extensive diffuse alveolar damage was observed, which was quite similar to sepsis or severe acute viral pneumonia, such as the fatal cases of COVID-19 (Sadegh Beigee et al., 2020) or in HIV/AIDS immune deficient patients with bacterial infections (de Matos Soeiro et al., 2008). Alveolar diffuse damage is occasionally reported in miliary TB, which is a condition similar to sepsis (Kim et al., 2003).

It is known that an imbalance of Th17/Tregs is associated with poor prognosis in acute pulmonary inflammation (Cheung et al., 2011), and it has been demonstrated that Treg cells constrain pathogenic Th17 cells (Fletcher et al., 2009). We observed numerous IL-17 cells, particularly in the inflammatory infiltrate associated with areas of alveolar diffuse damage, which suggest an imbalance of Th17/Treg cells in these areas. This focal circumscribed location of a high number of IL-17 cells could explain the similar expression of IL-17 between Treg cells depleted and control animals that showed extensive pneumonia with numerous Treg cells, determined by RT-PCR in whole lung homogenates. A similar pattern was seen in the expression of the pro-inflammatory cytokine TNF-α and IL-22 which are significant participants in this kind of pulmonary lesion (Eyerich et al., 2017). Diffuse alveolar damage is characterized by hyaline eosinophilic membranes deposited on the surface of the alveolar epithelium. These membranes are essentially constituted by fibrin, which was clearly evidenced by its positive staining with the phosphotungstic acid hematoxylin technique. Capillary endothelium in areas of diffuse alveolar damage suffers changes that permit leukocyte recruitment and activation, as well as plasma proteins exudation. Among the most important changes is the overexpression of adhesion molecules, such as PECAM-1, GPIV, P-selectin, and VCAM-1. In areas of diffuse alveolar damage, all these molecules were demonstrated by strong immunohistochemistry positivity in capillary endothelium and some inflammatory cells. PECAM-1 is highly expressed in activated endothelial cells and in platelets, monocytes, neutrophils, T cells, and B cells; it has a crucial role in capillary morphogenesis, migration, and junctional development (Privratsky and Newman, 2014). PECAM-1 has anti-inflammatory and pro-inflammatory functions (Carrithers et al., 2005; Sugimoto et al., 2008; Privratsky and Newman, 2014). Here, we observed more expression of PECAM-1, particularly in endothelial cells and recruited leucocytes in the areas of alveolar damage, so this molecule may contribute to this type of lesion through cell recruitment and fluid exudate production. Major glycoprotein of platelets (CD36) is a scavenger receptor expressed by platelets, macrophages, endothelial cells, and smooth muscle cells. CD36 participates in tumor maintenance through its expression in intratumoral Treg cells by CD36-dependent metabolic adaptation (Wang et al., 2020) and also participates in inflammation and sterile inflammation *via* TLR 4 and 6 activations (Stewart et al., 2010; Xu et al., 2018). We observed an increase of CD36 positive cells in mice with 5186 strain infection treated with anti-CD25; the high expression of CD-36 can enhance a pro-inflammatory environment favoring alveolar acute damage. Another adhesion molecule that we observed increased in mice infected with hypervirulent strain and treated with anti-CD25, was P-selectin. This molecule is expressed on activated endothelium and platelets, and its ligands PSGL1 and PSL2 are expressed in leucocytes, participating in T-cell recruitment during antigen stimulation (Carlow et al., 2018). We observed P-selectin expression in endothelial cells and interestingly in macrophages, which indicates endothelium activation that enhances inflammatory cells recruitment. Recently there was reported the importance of homeostasis of Treg cells and platelets *via* CD40L-CD40 and PSGL1-P-Sel interaction, the interruption of these pathways leads to hyperinflammation (Rupp et al., 2021), which partially can explain the excessive inflammatory response when Tregs cells were depleted during infection with hypervirulent MTB strain. Finally, we evaluated VCAM-1, which is an Ig-like adhesion molecule expressed on endothelial cells induced by inflammatory cytokines that play a critical role in leucocyte recruitment during acute or chronic inflammation (Chen and Massagué, 2012). VCAM-1 overexpression was associated with lung inflammation (Park et al., 2021), and it is highly expressed on endothelial cells located in the areas of alveolar damage developed in mice treated with anti-CD25. Thus, the high expression of all these adhesion molecules in lungs from mice infected with strain 5186 and treated with anti-CD25 should contribute to the observed diffuse alveolar damage; however, more studies are necessary to elucidate their real contribution in this type of tissue damage, which should also be related with devitalization of the lung tissue and the immune response considering the very high bacillary loads that were found in these mice.

Small and occasional areas of alveolar damage were seen in mice infected with highly virulent strain 5186 treated with both drugs to block the activity of IDO and HO-1; the percentage of pneumonic areas was lesser in treated mice than in control animals, but in contrast with control animals, treated mice showed in the pneumonic areas extensive areas of necrosis. On the other hand, HO-1 metabolites also have cytoprotective proprieties, for example, which protect against free radical-mediated immunopathology, limiting the damage during TB infection (Chinta et al., 2018), there is evidence of the importance of HO-1 to protect from necrosis during TB infection that was observed in mice infected with hypervirulent strain and blocking HO-1 and IDO activity. The lungs of these treated animals showed significantly lower expression of TGF-β than control mice, suggesting that one of the factors related to necrosis was the suppression of TGF-β, which is a significant anti-inflammatory factor that participates in the control of excessive inflammation during late infection in this experimental model (Hernández-Pando et al., 2006) and human TB (Toossi and Ellner, 1998).

In conclusion, our results suggest that Treg cells delay the recruitment of effector cells and balance the inflammatory response during early infection with mild virulence or hypervirulent MTB strain, their depletion increases some proinflammatory cytokines and neutrophil recruitment that reduce the bacillary load but is not enough to change the final course of the disease. IDO and HO-1 activities facilitate bacterial growth during late pulmonary TB when the infection is produced by mild virulence MTB. It seems that Treg cells and the enzymes IDO and HO-1 are crucial to suppress excessive inflammation, preventing diffuse alveolar damage or necrosis when the infection is produced by a highly virulent strain. Thus, one important factor that contributes to the protective or detrimental immune regulation activity is the infection phase and mycobacterial level of virulence.

## Data availability statement

The original contributions presented in the study are included in the article/**Supplementary Material**. Further inquiries can be directed to the corresponding author.

## Ethics statement

The animal study was reviewed and approved by Ethical Committee for Experimentation on Animals of the National Institute of Medical Sciences and Nutrition (INCMNSZ).

## Author contributions

VL-O and RH-P contributed to the background work. VL-O and RH-P conceived experiments. VL-O performed, organized, and analyzed the results. YR-M, AO-C and SH-B performed the experiments. DM-E and JB-P contributed to mice infection procedures. RS contributed to the production of anti-CD25 and flow cytometry design. All authors contributed to the article and approved the submitted version.
