# Supplementary material for: Beneficial or detrimental activity of regulatory T cells, indoleamine 2,3-dioxygenase, and heme oxygenase-1 in the lungs is influenced by the level of virulence of Mycobacterium tuberculosis strain infection
Source: Front Cell Infect Microbiol. 2023 May 22;13:1105872. doi: 10.3389/fcimb.2023.1105872 (PMC10239976; doi:10.3389/fcimb.2023.1105872)
Supplement: Supplementary file 1 [file DataSheet_1.docx]

Supplementary Material

**Beneficial or detrimental activity of regulatory T cells, indoleamine 2,3-dioxygenase and heme oxygenase-1 in the lungs is influenced by the level of virulence of *Mycobacterium tuberculosis* strain infection**

**Vasti Lozano-Ordaz, Yadira Rodriguez-Miguez, Angel E Ortiz-Cabrera, Sujhey Hernandez-Bazan, Dulce Mata-Espinosa, Jorge Barrios-Payan, Rafael Saavedra, Rogelio Hernandez-Pando^*^**

***Correspondence:**

**Rogelio Hernandez-Pando**

[rhdezpando@hotmail.com](mailto:rhdezpando@hotmail.com)

## Supplementary Figures


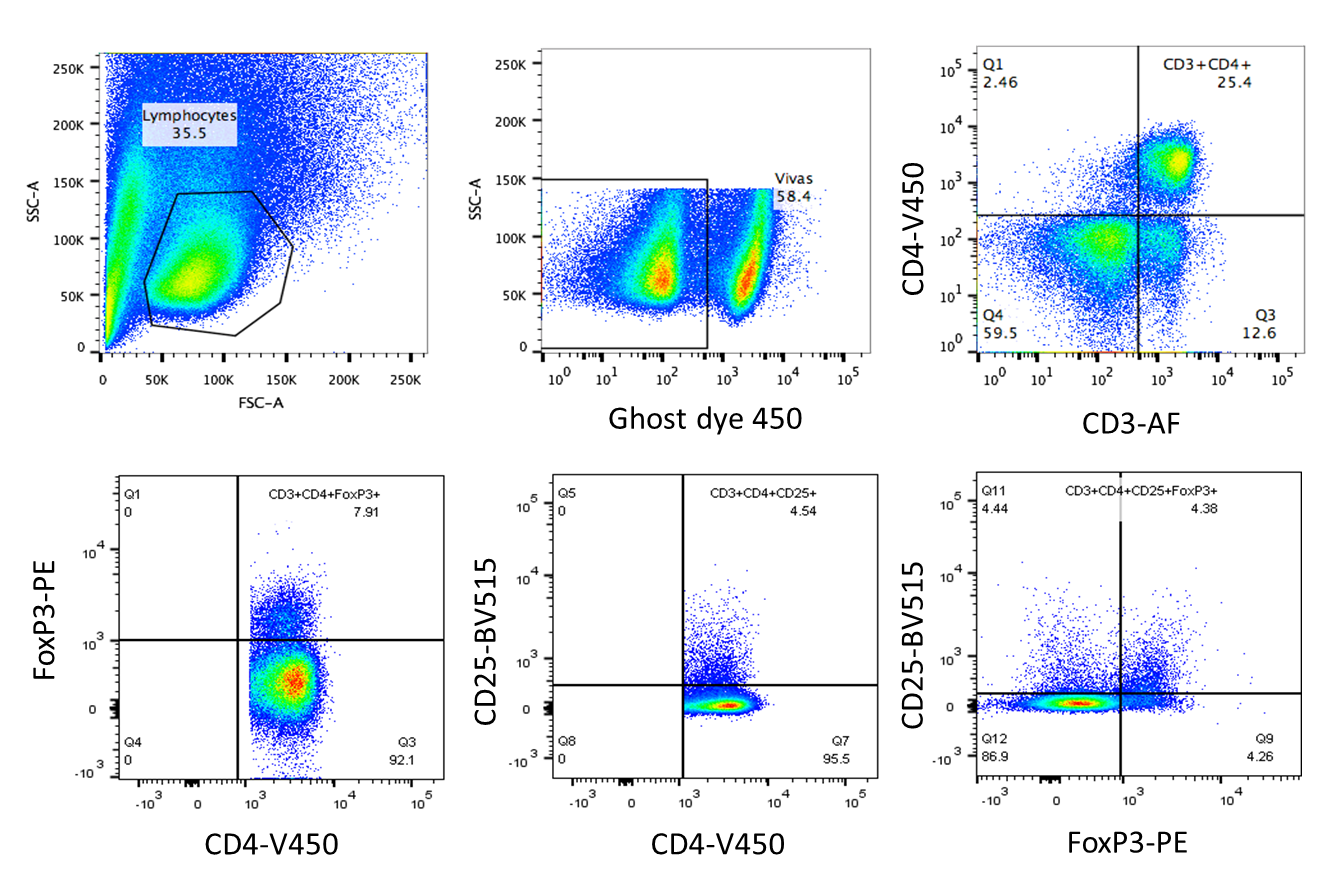


**Supplementary Figure 1. Flow cytometry strategy analysis of Treg cells.** There are representatives plots from 5186 infected mice lung, to show flow cytometry analysis. Lungs from 3 mice per group/time were disaggregated and stained. Lymphocytes zone was selected by size and granularity, then CD3+CD4+ were selected from alive cells. Finally, expression of FoxP3 and CD25 was used to identify Tregs cells.


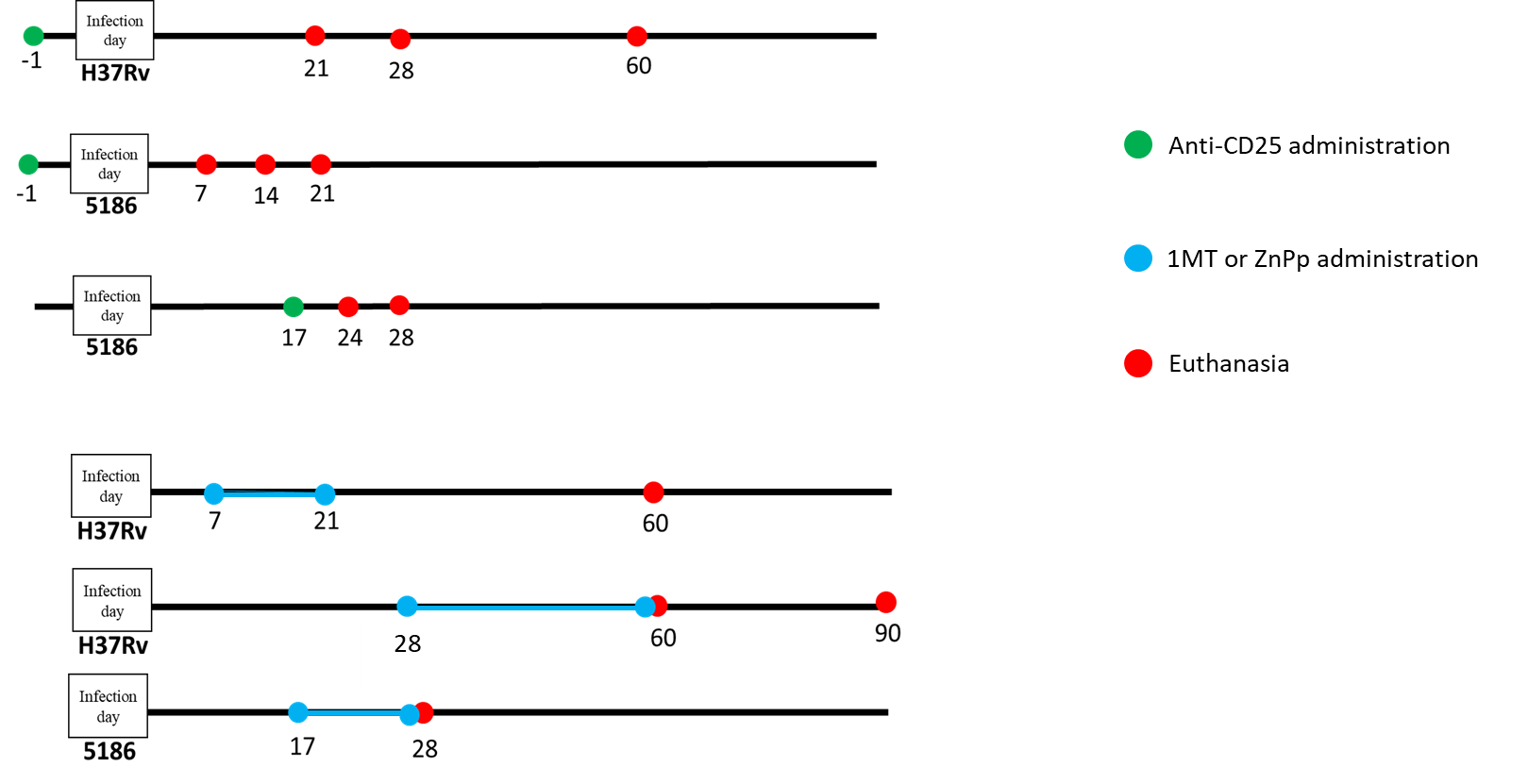


**Supplementary Figure 2. Timeline for infection and treatment.** Points for treatments and euthanasia were selected according to development disease with different Mycobacterium tuberculosis strain. Each dot represents days postinfection, continuous blue line, indicates daily treatment.


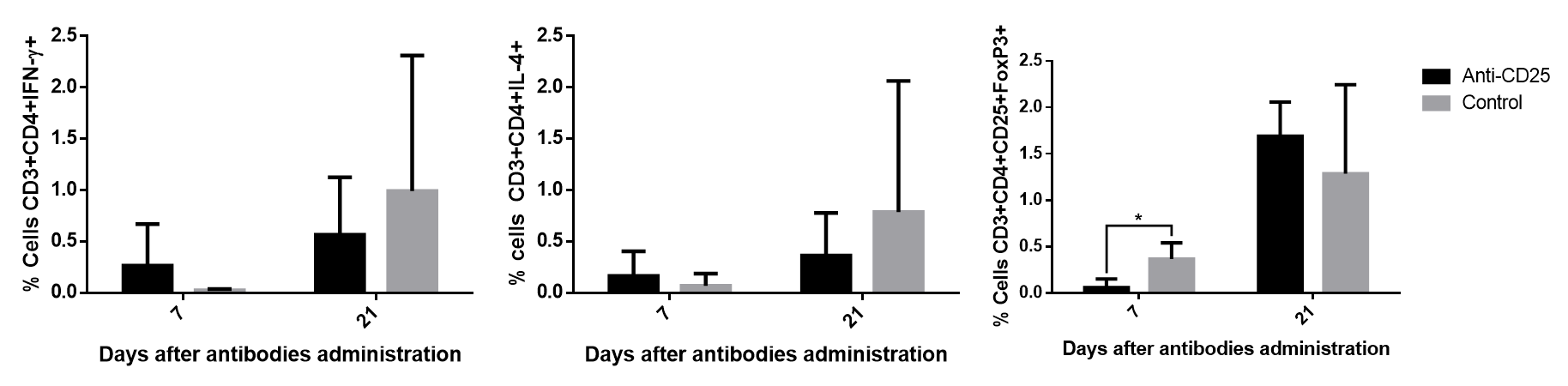


**Supplementary Figure 3. Percentages of T cells during anti-CD25 administration.** Representatives plots from flow cytometry analysis. Lungs from each mice group were disaggregated and stained. Lymphocytes zone was selected by size and granularity, then CD3+CD4+ were selected from alive cells. Finally, expression of FoxP3 and CD25 was used to identify Tregs cells.


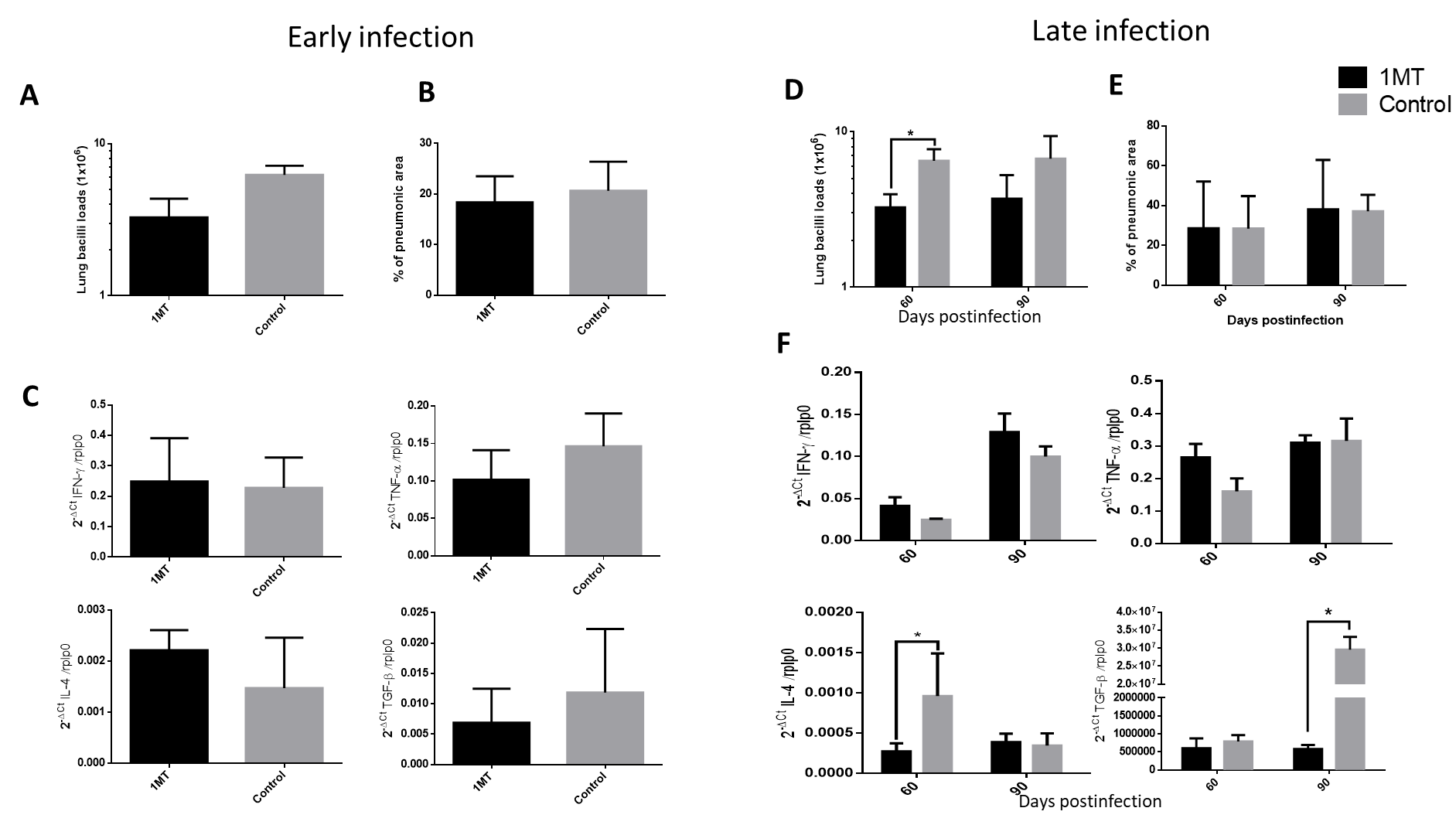


**Supplementary Figure 4. The effect of suppress IDO during early and late pulmonary infection with mild virulence strain.** A) The inhibitor 1MT was administrated during the second and third week of infection with strain H37Rv (early infection) and mice were euthanize at two months of infection, right lungs were used to determine bacilli loads. B) Left lungs were perfused with absolute ethylic alcohol and embedded in paraffin, tissue sections stained with hematoxylin/eosin were used to determine the percentage of lung area affected by pneumonia. C) The lungs of other group of infected mice from 1MT treated and non-treated animals were used to isolate total RNA and the expression of the indicated cytokines were determined by RT-PCR. D) 1MT was administered since the first month of the infection and during two months (late infection), euthanizing mice at 60 and 90 days after infection, the right lungs were used to determine bacillary burdens. The left lungs were used for histology analysis and determination of the percentage of pneumonia by automated morphometry (E). The lungs of other group of mice were used to isolate total RNA and determine the indicated cytokines by RT-PCR (F). Bars represent the means and standard deviation of three mice per time point, asterisk represent statistical difference (p<0.05 unpaired t-test).


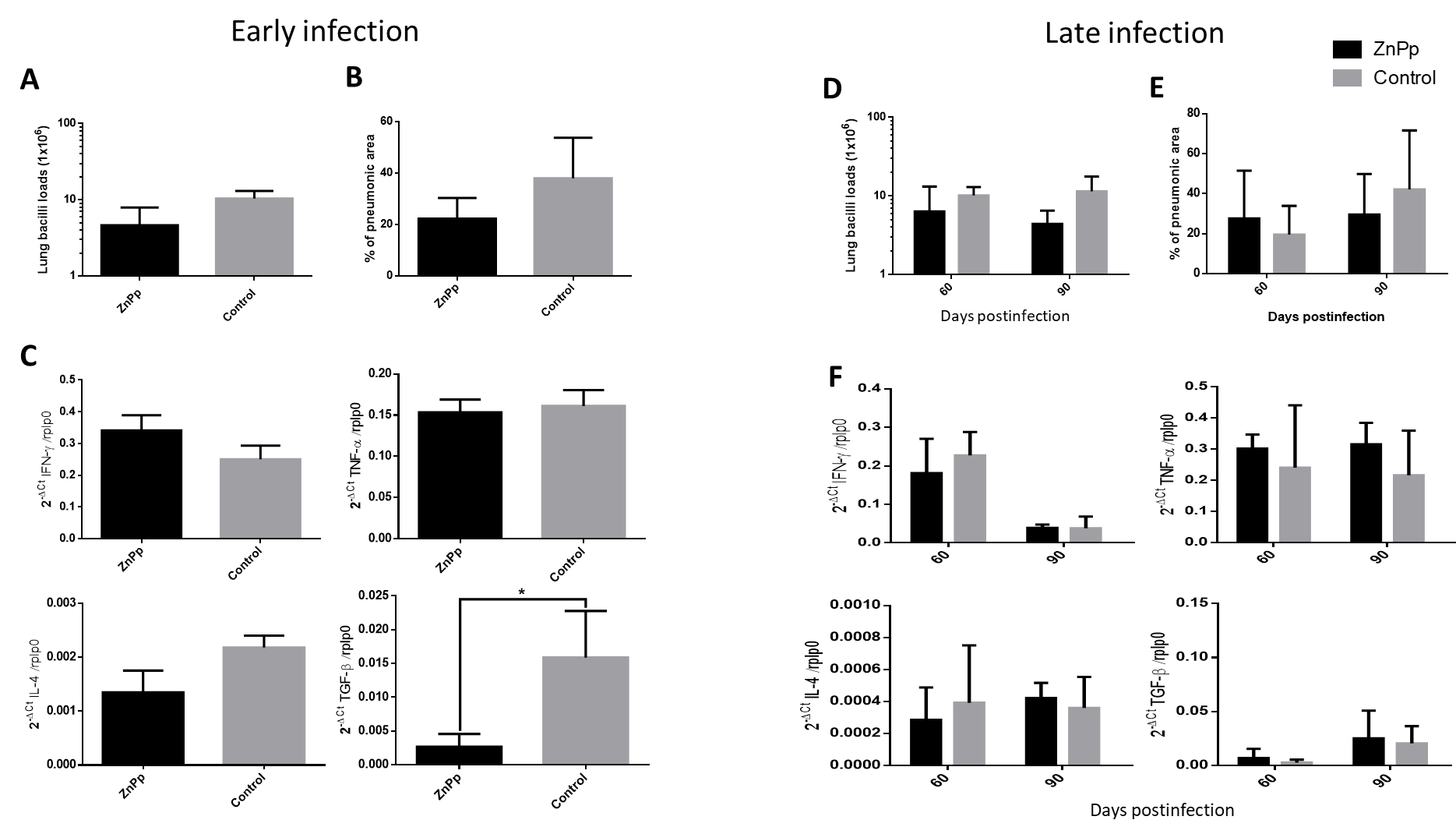


**Supplementary Figure 5. The effect of suppress HO-1 during early and late pulmonary infection with mild virulence mycobacteria strain**. A) The inhibitor ZnPp was administrated daily during the second and third week of infection with strain H37Rv (early infection) and mice were euthanize at two months of infection, right lungs were used to determine bacilli loads. B) Left lungs were prepared to get histological sections that were used to determine the percentage of lung area affected by pneumonia. C) The lungs of other group of infected mice from ZnPp treated and non-treated animals were used to isolate total RNA and the expression of the indicated cytokines were determined by RT-PCR. D) ZnPp was administered daily since the first month of the infection and for two months (late infection), euthanizing mice at 60 and 90 days after infection, the right lungs were used to determine bacillary burdens. The left lungs were used for determination of the percentage of pneumonia by automated morphometry (E). The lungs of other group of mice were used to determine the expression of the indicated cytokines by RT-PCR (F). Bars represent the means and standard deviation of three mice per time point, asterisk represent statistical difference (p<0.05 unpaired t-test).
